# Enhancing Effects of the Cyano Group on the C-X∙∙∙N Hydrogen or Halogen Bond in Complexes of X-Cyanomethanes with Trimethyl Amine: CH_3−n_(CN)_n_X∙∙∙NMe_3_, (n = 0–3; X = H, Cl, Br, I)

**DOI:** 10.3390/ijms231911289

**Published:** 2022-09-25

**Authors:** Rubén D. Parra, Sławomir J. Grabowski

**Affiliations:** 1 Department of Chemistry and Biochemistry, DePaul University, Chicago, IL 60614, USA; 2 Polimero eta Material Aurreratuak: Fisika, Kimika eta Teknologia, Kimika Fakultatea, Euskal Herriko Unibertsitatea UPV/EHU, Donostia International Physics Center (DIPC), P.O. Box 1072, 20080 Donostia, Spain; 3 Ikerbasque, Basque Foundation for Science, 48011 Bilbao, Spain

**Keywords:** halogen bond, hydrogen bond, density functional theory, ab initio calculations

## Abstract

In this paper, density functional theory and wave function theory calculations are carried out to investigate the strength and nature of the intermolecular C-X∙∙∙N bond interaction as a function of the number of cyano groups, CN, in the X-bond donor while maintaining the X-bond acceptor as fixed. Specifically, complexes of X-cyanomethanes with trimethyl amine CH_3−n_(CN)_n_X∙∙∙NMe_3_ (n = 0–3; X = H, Cl, Br, I) are used as model systems. Geometrical parameters and vibrational C-X-stretching frequencies as well as interaction energies are used as relevant indicators to gauge hydrogen or halogen bond strength in the complexes. Additional characteristics of interactions that link these complexes, i.e., hydrogen or halogen bonds, are calculated with the use of the following theoretical tools: the atoms in molecules (AIM) approach, the natural bond orbital (NBO) method, and energy decomposition analysis (EDA). The results show that, for the specified X-center, the strength of C-X∙∙∙N interaction increases significantly and in a non-additive fashion with the number of CN groups. Moreover, the nature (noncovalent or partly covalent) of the interactions is revealed via the AIM approach.

## 1. Introduction

A hydrogen bond, D-H∙∙∙A, is commonly understood as the attractive, non-covalent interaction between a hydrogen bond donor, D-H, and a hydrogen bond acceptor, A [1,2,3,4,5,6,7,8]. Likewise, a halogen bond, D-Hal∙∙∙A (Hal = F, Cl, Br, or I), is understood as the attractive, non-covalent interaction between a halogen bond donor, D-Hal, and a halogen bond acceptor, A [9,10,11,12,13,14,15,16]. Thus, a halogen bond could be considered in principle as an extension of the hydrogen bond concept. It has also been suggested that a hydrogen or a halogen bond could be thought of as a Lewis acid–base interaction, with the donor (D-H or D-Hal) as the Lewis acid, and the acceptor, A, as the Lewis base [13,14,15,16,17]. The distinctive characteristics of the hydrogen and halogen bond interactions, as well as their corresponding IUPAC (International Union of Pure and Applied Chemistry) definitions, have been provided recently [18,19].

Similarities and differences between hydrogen and the halogen bonds have been pointed out in a variety of studies [20,21,22,23,24,25]. For example, both interactions have been shown to be highly directional, although the halogen bond tends to be more linear than the hydrogen bond [26,27,28,29,30]. Additionally, both the hydrogen bond and the halogen bond have been shown to be highly tunable [14,15,16,17,31]. Tuning the strength of the hydrogen or halogen bond interaction for a fixed acceptor or Lewis base can be made through the interplay of a number of factors pertaining the D-H or the D-Hal donor group. With more than one type of halogen atom that can be used in D-Hal, the halogen bond also renders greater flexibility for fine-tuning compared with the hydrogen bond [32,33,34,35,36,37,38,39,40,41,42,43,44]. One factor that can be used to fine tune the D-H or the D-Hal donor ability is the electronegativity of the atom covalently bonded to hydrogen or halogen. In the case of the hydrogen bond donor, an increase in electronegativity reduces electron density in the hydrogen atom in D-H, making it more susceptible to interact with a Lewis base. Thus, C-H is considered a weak hydrogen bond donor when compared with either the O-H or the N-H hydrogen bond donor. In the case of the halogen bond donor ability of D-Hal, it has been found that both the size and the magnitude of the positive electrostatic potential (known as the σ-hole) of the halogen atom increases with the increasing electronegativity of the atom covalently bonded to the halogen atom in D-Hal. It is worth noting that the σ-hole on the halogen atom acts as the Lewis acid site that interacts with a suitable electron donor species acting as the Lewis base, and that the location of the σ-hole (opposite to the covalent D-Hal bond) results in the highly distinctive linearity of the halogen bond [9,32,44]. Further studies have demonstrated that the ability of a halogen atom to engage in halogen bonding correlates with the size of the σ-hole. Accordingly, the C-Hal is deemed a weak halogen bond donor when compared with either the O-Hal or the N-Hal donor. Moreover, it has also been found that for the same D, the size of the σ-hole increases with the size of the halogen (F ≪ Cl < Br < I). The correlation between the halogen atom size and the σ-hole adds flexibility for fine-tuning the halogen bond donor ability of D-Hal when compared with the fine-tuning of the hydrogen bond donor ability of D-H. Another factor that has been shown to influence hydrogen or halogen bond donor ability is the hybridization of the atom covalently bonded to the hydrogen (in D-H) or halogen atom in (D-Hal). Specifically, the donor ability of C-H or C-Hal tends to increase with the increasing *s*-character of the hybridized carbon, i.e., C(*sp*^3^) < C(*sp*^2^) < C(*sp*) [45,46,47,48]. The presence of electron-withdrawing groups have also been shown to enhance the donor ability of either the D-H or the D-Hal group. Thus, even a weak hydrogen (C-H) or halogen (C-Hal) bond donor with hybridized *sp^3^* carbon can increase its donor ability by adding suitable electron-withdrawing groups to the carbon atom [40,42,45].

In this work, the enhancing effect on the hydrogen bond or the halogen bond donor ability of methane or halomethane is investigated by sequentially adding up to three electron-withdrawing cyano groups, CN, as substituents on the hybridized *sp*^3^ carbon of the methane or the halomethane. Specifically, the strengthening of the hydrogen or the halogen bond is examined as a function of the number of cyano groups in the complexes CH_3−n_(CN)_n_X∙∙∙NMe_3_ (n = 0–3; X = H, Cl, Br, I). The following are the reasons for the choice of the CN group as a substituent: It is known as a strong electron-withdrawing substituent that also possesses simple structure. Moreover, this group has not been analyzed extensively as a factor influencing halogen bond strength, and not as extensively as fluorine and other halogens, for example.

For convenience, the hydrogen or the halogen bond interaction studied in this work is jointly represented as C-X∙∙∙N. Moreover, the relatively strong and neutral Lewis base trimethyl amine, NMe_3_, is used throughout as the halogen bond acceptor. Lastly, in this work, the correlation between the halogen bond strength and the halogen atom size is also examined. The choice of small Lewis acid (CH_3−n_(CN)_n_X) and Lewis base (NMe_3_) units allow us to apply various theoretical tools and consequently to analyze different characteristics of interactions. The particular attention is paid here on the comparison between hydrogen and halogen-bonded systems. The competition between these interactions is often observed in crystal structures; thus, the results presented here allow us to understand the influence of these interactions on the arrangement of molecules and ions in crystals.

## 2. Computational Details

Geometry optimizations, frequency calculations, and interaction energies were carried out using the GAUSSIAN 16 program [49]. Geometry optimizations for all complexes, CH_3−n_(CN)_n_X∙∙∙NMe_3_ (n = 0–3; X = H, Cl, Br, I) and constituent monomers were first carried out using the ωB97X-D [50] density functional method and the aug-cc-pVTZ-PP [51] basis set for the iodine atom and the aug-cc-pVTZ [52,53,54,55] basis set for all other atoms. Corresponding frequency calculations demonstrated that the ωB97X-D-optimized geometries were minimum energy structures with no imaginary frequencies. Subsequent geometry optimizations for all monomers and complexes were carried out using the MP2 [56,57] method with both the aug-cc-pVTZ-PP and the aug-cc-pVDZ-PP [58] basis sets for the iodine atom and the corresponding aug-cc-pVTZ or aug-cc-PVDZ basis sets for all other atoms. The MP2 geometries optimized with the smaller of the two basis sets (aug-cc-pVDZ-PP, aug-cc-pVDZ) were shown to be minimum energy structures with no imaginary frequencies calculated at the same level of theory.

The ωB97X-D geometries of complexes optimized with the larger basis set (aug-cc-pVTZ-PP, aug-cc-pVTZ) were used for all subsequent analyses including interaction energy, NBO, and AIM analyses. In particular, topological features of electron density according to the theory of atoms in molecules (AIM) of Bader [59] were obtained with the AIMALL [60] software package. MP2 interaction energies were obtained for all complexes and corrected for basis set superposition error, BSSE, using the counterpoise method [61]. BSSE-corrected interaction energies were also obtained with the CCSD(T) [62] method for the X = H- or X = I-containing systems.

The BP86-D3/TZ2P calculations were carried out with the ADF 2019.302 program codes [63] for the decomposition of interaction energies [64,65] for the ωB97X-D-optimized dimer complexes described previously. That is, the BP86 functional [66,67] with Grimme dispersion corrections [68] was applied, and for all elements the uncontracted Slater-type orbitals (STOs) as basis functions with triple-ζ quality [69] were applied. Relativistic scalar ZORA corrections [65] were applied for species containing heavier atoms (Br and I).

The NBO method [70,71] was used to calculate the atomic charges, the energies of orbital–orbital interactions, the Wiberg bond indices [72], and the natural binding indices. The NBO 6.0 program [73] implemented in the ADF2019 set of codes [63] was applied to perform NBO calculations.

The calculations of interaction energies were performed with the splitting of the complexes CH_3−n_(CN)_n_X∙∙∙NMe_3_ into the CH_3−n_(CN)_n_X and NMe_3_ units. The same splitting was carried out in the decomposition of interaction energies as well as in other calculations where interactions between Lewis acid and Lewis base units were considered.

## 3. Results and Discussion

### 3.1. Geometries and Vibrational Frequencies

**Figure 1:**
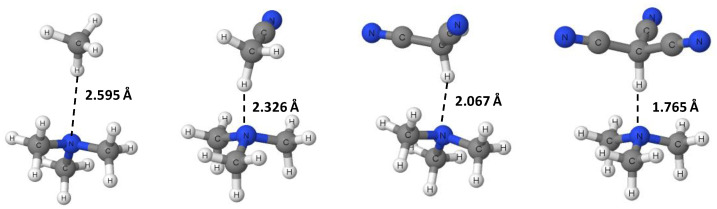
ωB97X-D/aug-cc-pVTZ-optimized geometries of the CH_3−n_(CN)_n_H∙∙∙NMe_3_ (n = 0–3) complexes.

**Figure 2:**
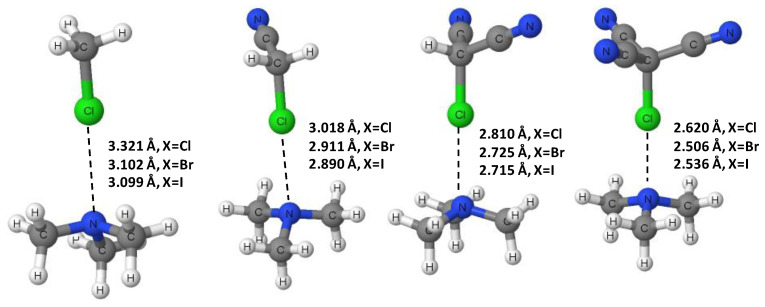
ωB97X-D/aug-cc-pVTZ-optimized geometries of the CH_3−n_(CN)_n_Cl∙∙∙NMe_3_ (n = 0–3) complexes; X∙∙∙N distances for X = Cl, Br, I are presented in the figure.

Optimized geometries for all CH_3−n_(CN)_n_X∙∙∙NMe_3_ (n = 0–3; X = H, Cl, Br, I) complexes were first obtained with the ωB97X-D/aug-cc-pVTZ level of theory. Harmonic vibrational frequency calculations, at the same level of theory, demonstrated that all the optimized geometries were minima in their respective potential energy surfaces (no imaginary frequencies present). Geometry optimizations and corresponding frequency calculations at the MP2/aug-cc-pVDZ level of theory also resulted in minimum energy structures for all complexes with no imaginary frequencies. Lastly, the MP2/aug-cc-pVDZ-optimized geometries were used as initial guess geometries for further geometry optimizations at the MP2/aug-cc-pVTZ level. Frequency calculations at the MP2/aug-cc-pVTZ were generally not possible given our computer capabilities, and thus were not performed. It should be noted that, in all calculations, either the aug-cc-pVDZ-PP or the aug-cc-pVTZ-PP basis set was used for the iodine atom, where applicable. Figure 1 and Figure 2 show the ωB97X-D/aug-cc-pVTZ-optimized geometries of the CH_3−n_(CN)_n_H∙∙∙NMe_3_ and CH_3−n_(CN)_n_Cl∙∙∙NMe_3_ complexes; the X∙∙∙N distances are also presented there for all complexes, X = H, Cl, Br, I. The corresponding relevant geometrical parameters for each complex at all levels of theory can be found in Table 1, Table 2, Table 3 and Table 4. Accordingly, the inspection of Table 1, Table 2, Table 3 and Table 4 shows that the ωB97X-D/aug-cc-pVTZ level of theory predicts C-X bond lengths and intermolecular X∙∙∙N distances that are smaller and longer, respectively, than the corresponding results predicted in the MP2/aug-cc-pVDZ level of theory. Interestingly, the MP2/aug-cc-pVTZ results are closer to those of the ωB97XD/aug-cc-pVTZ level of theory for the C-X covalent bond lengths and to those of the MP2/aug-cc-pVDZ level of theory for the hydrogen or halogen bond distances and angles, X∙∙∙N and C-X∙∙∙N, respectively.

**Figure 3:**
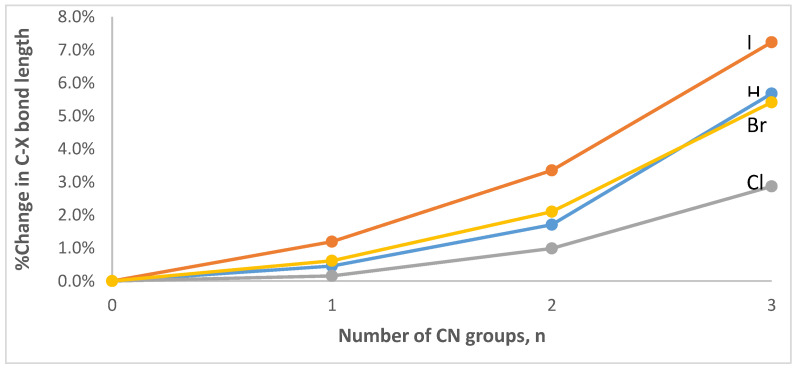
Percent change in the covalent C-X bond as a function of the number of CN groups in the CH_3−n_(CN)_n_X∙∙∙NMe_3_ (X = H, Cl, Br, or I; n = 0–3) complexes optimized at the ωB97X-D/aug-cc-pVTZ level of theory.

**Figure 4:**
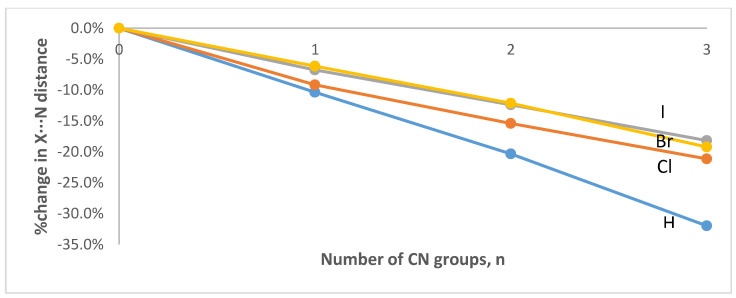
Percent change in the intermolecular X∙∙∙N distance as a function of the number of CN groups in the CH_3−n_(CN)_n_X∙∙∙NMe_3_ (X = H, Cl, Br, or I; n = 0–3) complexes optimized at the ωB97X-D/aug-cc-pVTZ level of theory.

An inspection of Table 1 shows that, at all levels of theory, the covalent C-H bond length in CH_3−n_(CN)_n_H∙∙∙NMe_3_ increases nonlinearly with the number of CN groups, relative to the bond length for the system with no CN groups, n = 3. For example, at the ωB97X-D/aug-cc-pVTZ level, the C-H bond increases by only 0.5% when n = 2, but then it increases more substantially when n = 1 (1.7%) and when n = 0 (5.7%). Concomitant with the C-H elongation there is a nonlinear reduction in the hydrogen bond distance, H∙∙∙N, with the number of CN groups that is proportionally larger than the accompanying increase in the C-H bond length. Thus, at the ωB97X-D/aug-cc-pVTZ level, the H∙∙∙N distance is decreased by 10.4%, 20.3% and 32.0% for n = 2, 1, and 0, respectively. Lastly, the C-H∙∙∙N angle remains far from linear until all three CN groups are present. Overall, the geometrical changes suggest an important strengthening of the H∙∙∙N hydrogen bond resulting from the cumulative electron-withdrawing effects of the CN groups. An inspection of Table 2, Table 3 and Table 4 reveals that the effects of the CN groups on the C-X bond lengths and the X∙∙∙N halogen bond distances qualitatively mirror those in the hydrogen bond complexes, i.e., X = H. A graphical representation of the effects of the CN groups at the ωB97X-D/aug-cc-pVTZ level can be seen in Figure 3 (for the C-X bond lengths) and in Figure 4 (for the X∙∙∙N interaction distances). It is worth noting that the halogen bond angles are linear for both X = Br and I, regardless of the number of CN groups. For X = Cl, the halogen bond angle increases from quasilinear 169° (n = 3) to linear 180° (n = 0).

**Figure 5:**
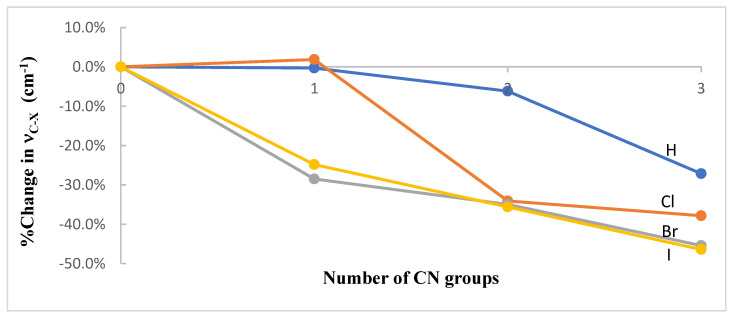
Percent change in the harmonic stretching frequency of the C-X bond, ν_C-X_, as a function of the number of CN groups in the CH_3−n_(CN)_n_X∙∙∙NMe_3_ (X = H, Cl, Br, or I; n = 0–3) complexes optimized at the ωB97X-D/aug-cc-pVTZ level of theory.

Changes in the strength of the hydrogen or halogen bond interactions revealed through geometrical parameters are also revealed in pertinent vibrational frequencies. Specifically, for any given complex, the elongation of the C-X bond length with the number of CN groups brings about a reduction in the stretching C-X vibrational frequency, ν_C-X_, as can be seen in Table 5 for the frequencies calculated at both the ωB97X-D/aug-cc-pVTZ and the MP2/aug-cc-pVDZ levels of theory. In particular, the percent changes in ν_C-X_ as a function of the number of CN groups for the CH_3−n_(CN)_n_X∙∙∙NMe_3_ systems at the ωB97X-D/aug-cc-pVTZ level are displayed in Figure 5.

### 3.2. Intermolecular Interaction Energies and AIM Analyses

**Figure 6:**
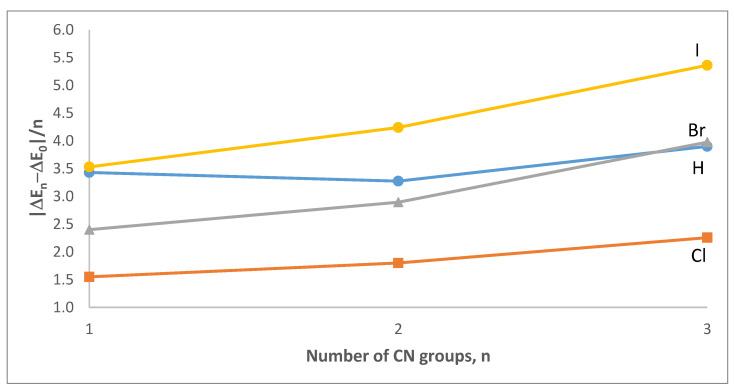
Changes in the magnitude of the interaction energy per CN group added in the CH_3−n_(CN)_n_X∙∙∙NMe_3_ (X = H, Cl, Br, or I; n = 0–3) dimer complexes. Values calculated at MP2//ωB97X-D/aug-cc-pVTZ level of theory.

Table 6 displays the BSSE-corrected interaction energy associated with the formation of each complex calculated at the MP2/aug-cc-pVTZ level using the geometries optimized at the ωB97X-D/aug-cc-pVTZ level of theory. An inspection of Table 6 shows that, even in the absence of a CN group, all complexes are stabilized by the C-X∙∙∙N interaction. In particular, the strength of the interaction follows the order H < Cl < Br < I when n = 0. However, H and Cl swap places when n = 1–3. Table 6 also shows that the increase in the magnitude of the interaction energy for any given complex increases nonlinearly with the number of CN groups. The rather substantial and non-additive increase in the interaction energy per CN group is presented in Figure 6. Specifically, the magnitude of the interaction energy per CN group increases with the number of CN groups for all halogen bonds. This increase also correlates with the size of the halogen. For the hydrogen bond, the magnitude of the interaction energy per CN group increases with one CN group but then shows a small decline with two CN groups followed by a somewhat large increase with three CN groups. It is worth noting that the increases in the magnitude of the interaction energies for the hydrogen bonds are consistently larger than in the chlorine bonds.

Table 7 displays the BSSE-corrected interaction energies associated with the formation of the C-H∙∙∙N and C-I∙∙∙N links in complexes calculated at the CCSD(T)/aug-cc-pVTZ level using the MP2/aug-cc-pVTZ-optimized geometries. An inspection of Table 7 shows similar qualitative trends in interaction energies as a function of the CN groups, as seen at the corresponding MP2 level. The magnitude of the interaction energies in all cases, however, appears somewhat overestimated at the MP2 level when compared with the corresponding CCSD(T) results.

The presence of a halogen (hydrogen) bond in each of the CH_3−n_(CN)_n_X∙∙∙NMe_3_-optimized complexes was further confirmed by the topology of the corresponding electron density [24,74]. In particular, a bond critical point was found for each of the C-X∙∙∙N interactions along the path connecting the nitrogen atom with the related hydrogen (X = H) or halogen (X = Cl, Br, I). The strength and nature of the interaction was gauged by examining properties evaluated at the bond’s critical point. In particular, the electron densities, ρ_c_, evaluated at the C-X∙∙∙N bond critical points, are typically used as indicators of bond strength. Moreover, the total electron energy densities, H_c_, and their kinetic and potential energy density components, G_c_ and V_c_, respectively, can provide insight on the nature of the hydrogen or halogen bond. [24,74,75,76,77] Table 8 shows the relevant AIM parameters for the various complexes considered (X = H, Cl, Br, and I).

**Figure 7:**
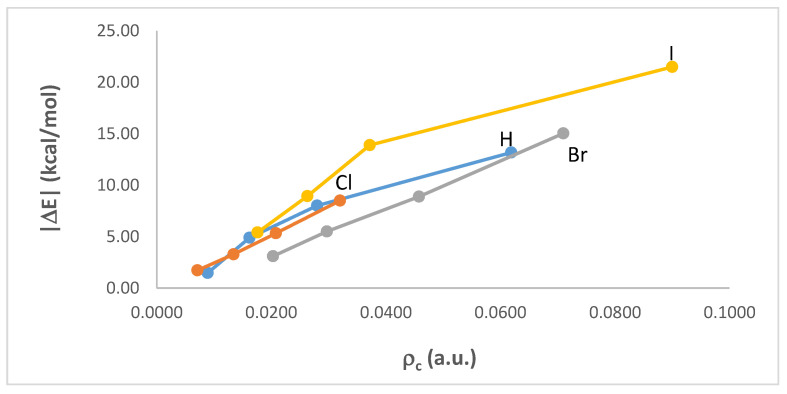
Correlations between ρ_c_ and the corresponding magnitude of interaction energies, |ΔE|, in the CH_3−n_(CN)_n_X∙∙∙NMe_3_ (X = H, Cl, Br, or I; n = 0–3) dimer complexes.

Inspection across Table 8 shows, for any given complex system, an increase in ρ_c_ with the number of CN groups, n. A closer examination of the data reveals a non-additive effect on ρ_c_. In the C-H∙∙∙N hydrogen bond interaction, for example, ρ_c_ increases by 0.0073 a.u. when n = 1, but more than twice this amount when n = 2 (0.0191 a.u.), and more than six times (0.0461 a.u.) when n = 3. Table 9 summarizes the non-additive changes in ρ_c_ per CN group for any given C-X∙∙∙N interaction. The strengthening of the C-X∙∙∙N bond, reflected in the non-additive increase in ρ_c_ with the number of CN groups, is further verified through the correlations found between ρ_c_ and the corresponding magnitude of interaction energies, |ΔE|, as shown in Figure 7. In general, for any X, the larger the magnitude of the interaction energy is, the larger the corresponding ρ_c_ is. More specifically, ρ_c_ and |ΔE| correlate linearly when X = Cl or Br, and nonlinearly when X = I or H.

Insight into the nature of the C-X∙∙∙N bond in a given complex is gained by examining the sign of the corresponding electron energy density at the bond’s critical point (H_c_) [24,74,75,76,77]. Indeed, a positive sign of H_c_ would correspond to a closed-shell intermolecular interaction, but a negative sign would correspond to a partly covalent one. Further confirmation on the nature of the interaction can be found in the absolute ratio of the kinetic, G_c_, and potential, V_c_, electron energy density components of H_c_ |G_c_/V_c_|. Accordingly, if |G_c_/V_c_| > 1, then the interaction is noncovalent. On the other hand, if 0.5 < |G_c_/V_c_| < 1, then the nature of the interaction is deemed to be partly covalent. Accordingly, Table 8 shows that the nature of the C-H∙∙∙N hydrogen bond transition from a closed-shell noncovalent interaction into a partly covalent one when the number of CN groups is two. Likewise, the transition to the partly covalent interaction in C-Br∙∙∙N occurs when n = 2. The transition to partly covalent for the C-Cl∙∙∙N halogen bond interaction occurs only when there are three CN groups (Table 8). In turn, Table 8 reveals that only one CN group suffices for C-I∙∙∙N halogen bond interaction to exhibit a partly covalent nature. In general, the |G_c_/V_c_| ratio decreases with the number of CN groups in accord with a systematic strengthening of the C-X∙∙∙N bond interaction brought about by the electron-withdrawing CN groups.

### 3.3. Energy Decomposition Analysis

In the decomposition scheme [63,64], the total interaction energy is partitioned according to the equation given below.

ΔE_int_ = ΔE_elstat_ + ΔE_Pauli_ + ΔE_orb_ + ΔE_disp_ (1)

The term ΔE_elstat_ is usually attractive and it corresponds to the quasi-classical electrostatic interaction between the unperturbed charge distributions of atoms. The Pauli repulsion, ΔE_Pauli_, is the energy change associated with the transformation from the superposition of the unperturbed electron densities of the isolated fragments to the wave function that properly obeys the Pauli principle through the antisymmetrization and renormalization of the product wave function. The orbital interaction, ΔE_orb_, corresponds to the charge transfer and polarization phenomena, i.e., to electron charge shifts resulting from the complex formation; the dispersion interaction energy term, ΔE_disp_, is also included (Equation (1)). In agreement with the convention adopted in the majority of studies, the attractive energy terms are negative while the Pauli repulsion is positive. Table 10 presents the results of the decomposition of the energy of interaction (Equation (1)) for complexes analyzed in this study.

The total BP86-D3/TZ2P interaction energies, ΔE_int_’s, are also inserted in Table 10. The latter energies are in good agreement with those calculated at the MP2/aug-cc-pVTZ level; for the complexes analyzed here, the linear correlation occurs between the MP2 and DFT(BP86-D3) interaction energies (R = 0.99). As pointed out earlier for the CH_n_(CN)_3−n_X∙∙∙N(CH_3_)_3_ complexes, the strength of interaction increases, i.e., |ΔE_int_| value increases, for the specific substituent X with the increase in the number of CN substituents. On the other hand, for the specific number of CN substituents in the complex the strength of interaction increases (|ΔE_int_|) with the increase in the atomic number of the halogen atom (X). Additionally, the interactions in CH_3−n_(CN)_n_H∙∙∙N(CH_3_)_3_ complexes are stronger than the corresponding interactions in the CH_3−n_(CN)_n_Cl∙∙∙N(CH_3_)_3_ systems. This means that the C-H∙∙∙N hydrogen bonds are stronger than the C-Cl∙∙∙N halogen bonds. However, the other C-X∙∙∙N halogen bonds (X = Br, I) are stronger than the corresponding hydrogen bonds. This is in line with other studies where it was found that hydrogen bonds are stronger than halogen bonds if the chlorine atom is a Lewis acid halogen center, while for heavier halogens, in contrast, the halogen bonds are stronger than their hydrogen bond counterparts [21,22,23,24,25,26]. These findings are in agreement with those based on the DFT results that are described in the former section. However, there is only one exception: for the former DFT results, for systems without CN groups (n = 0) the chlorine bond is stronger than the corresponding hydrogen bond.

Concerning the interaction energy terms, some interesting observations can be made. For example, the corresponding ΔE_Pauli_, |ΔE_elstat_| and |ΔE_orb_| values increase with the number of CN groups in the following order of X substituents: H < Cl < Br < I; there are only slight disagreements sometimes. In the case of the increase in the |ΔE_disp_| value, the order is slightly different, the same as for the |ΔE_int_| values: Cl < H < Br < I. Table 10 also shows that the electrostatic term is the most important attractive contribution of the total interaction energy, followed by the orbital term and the dispersion interaction energy term. In other words, the following order of terms is observed: |ΔE_elstat_| > |ΔE_orb_| > |ΔE_disp_|. However, there are few exceptions that concern the weaker interactions: in the CH_3_H∙∙∙N(CH_3_)_3_ complex, the order of |ΔE_disp_| > |ΔE_elstat_| > |ΔE_orb_| is observed, while for the CH_2_CNH∙∙∙N(CH_3_)_3_ and CH_3_Cl∙∙∙N(CH_3_)_3_ complexes, this order is |ΔE_elstat_| > |ΔE_disp_| > |ΔE_orb_|. The latter three complexes are among those with the weakest interactions. It is worth noting that for these three complexes exhibiting “the unusual order”, the repulsion interaction energy term, ΔE_Pauli_, is the lowest in comparison with its value for the remaining complexes (lower than 8 kcal/mol). Interestingly, the CH_2_CNCl∙∙∙N(CH_3_)_3_ complex, with just one CN group, shows the same order of attraction interaction energy contributions as the majority of all other complexes.

One may expect that the most important electrostatic interaction for the majority of complexes analyzed here would be contrary to the covalent character of the links in these complexes. The orbital interaction, related to the electron charge shifts resulting from complexation, is often attributed to covalency [5,8,71,77,78]. However, in several studies, it was pointed out that the positive value of the Heitler–London interaction energy term, ΔE_H-L_ = ΔE_Pauli_ + ΔE_elstat_, indicates the at least the partial covalent character of the interaction [78]. A positive ΔE_H-L_ indicates that the electrostatic attraction cannot balance the Pauli repulsion and that the stabilization is possible due to the electron charge density shifts expressed by the ΔE_orb_ term. For all complexes analyzed here, the pertinent ΔE_H-L_ values are always positive, indicating the covalent character of the interactions.

It is known that all interaction energy terms increase (repulsive Pauli term and absolute values of the attractive terms) with the increase in the strength of the interaction. However, the |ΔE_orb_| increases faster than |ΔE_elstat_| and |ΔE_disp_| [78]. Hence, the |ΔE_elstat_|/|ΔE_orb_| ratio decreases with the increase in the strength of interaction, or more specifically, with the increase in the covalent character of interaction. It was found, for example, that for extremely strong hydrogen bonds this ratio is lower than unity [78]. Table 10 shows that, in the case of the complexes analyzed here, for the specified X substituent the |ΔE_elstat_|/|ΔE_orb_| ratio generally decreases with the increase in CN substituents. The lowest value of this ratio, of 1.1, occurs for the C(CN)_3_H∙∙∙N(CH_3_)_3_ and C(CN)_3_Cl∙∙∙N(CH_3_)_3_ complexes.

**Figure 8:**
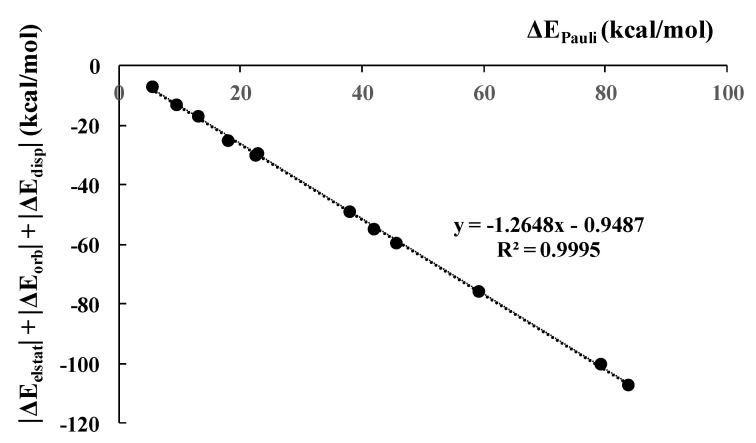
The linear correlation between the Pauli repulsion interaction energy and the sum of all attractive interaction energy terms for halogen-bonded dimers.

As mentioned above, all interaction energy terms increase with the increase in the strength of interaction (absolute values for attractive terms). Particularly, the attractive terms increase to compensate for the increase in the Pauli repulsion term [78]. Figure 8 presents an excellent linear correlation (R = 1.000) between the repulsion term and the sum of attractive terms if the halogen is the Lewis acid center, i.e., if the complexes are linked by halogen bonds. However, the hydrogen-bonded systems not presented in this figure are not so far from this linear correlation.

### 3.4. NBO Analyses

Table 11 presents various parameters calculated with the use of the NBO6 program. These parameters include the NBO atomic charges of X and N centers that are in contact. The nitrogen center which belongs to the N(CH_3_)_3_ Lewis base unit is always negative. The most negative charges of nitrogen occur for complexes linked by the CH∙∙∙N hydrogen bonds, between −0.34 au and −0.37 au. For the remaining complexes, this charge is located between −0.25 au and −0.34 au. Various situations are observed for the X charge of the Lewis acid species. The hydrogen center (X = H) is always positive, which may indicate the electrostatic character of the C-H∙∙∙N hydrogen bond. The same goes for iodine as the Lewis acid center: it is characterized by a positive charge. This is in line with other studies where it was found that the Lewis acid properties of halogen increase with the increase in its atomic number [27,79]. Similarly, the atomic charge for another heavier halogen, bromine, is positive, although less so than the corresponding iodine of the Lewis acid unit possessing the same number of CN substituents. There is one exception here: in the CH_3_Br∙∙∙N(CH_3_)_3_ complex, the bromine center is negative. In the case of the chlorine center, its charge is negative except for the CH(CN)_2_Cl∙∙∙N(CH_3_)_3_ complex, which shows a positive charge, albeit very close to zero.

**Figure 9:**
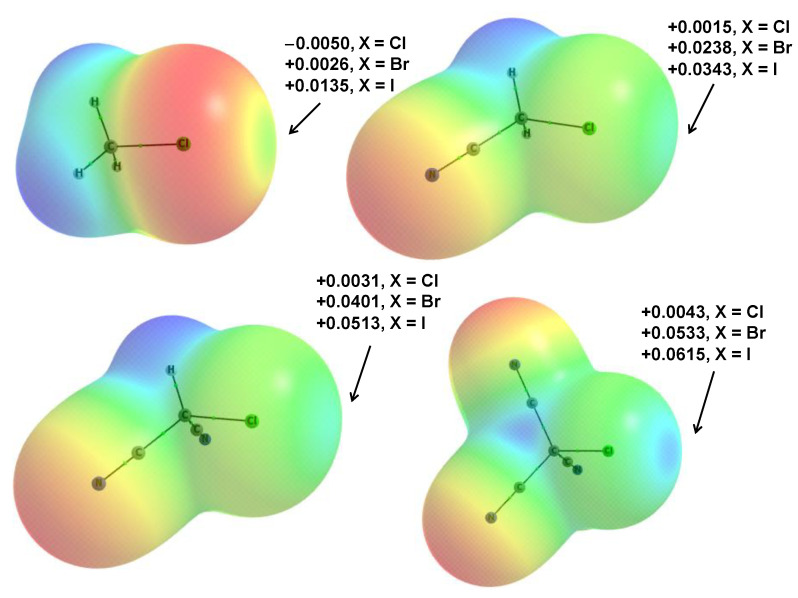
The EP surfaces of the CH_n-3_(CN)_n_Cl monomers. The EP maxima on the halogen σ-holes are listed (in au) for X = Cl, Br, I. The number of CN groups increases from 0 to 3.

It is worth noting, however, that electrostatic potentials are better indicators of the Lewis acidity–basicity than the charges [27,79]. The electrostatic potential (EP) surfaces of the CH_3−n_(CN)_n_Cl units acting as the Lewis acids in the complexes analyzed here are presented in Figure 9. These EP calculations were performed at the ωB97XD/aug-cc-pVTZ level. The EP surfaces correspond to the electron density of the 0.001 au; this electron density value was proposed by Bader and coworkers as corresponding approximately to the van der Waals spheres [80]. The EP values corresponding to the σ-holes at the halogen centers of all CH_3−n_(CN)_n_X units (X = Cl, Br, I) are presented in Figure 9. One can see that the EP value for the fixed halogen center increases with the increase in the number of CN substituents; similarly, for the fixed number of CN substituents, greater EP values are observed for a greater atomic number of the halogen center. The latter is in agreement with former studies where, for the same group elements, the EP value increased with the increase in atomic number [27,79]. One can see that the EP value refers approximately to the Lewis acid properties of the center considered. The EP value of the Lewis acid unit correlates often with the strength of interaction for complexes characterized by the same Lewis base units. For the halogen-bonded complexes analyzed in this study, a good second-order polynomial correlation is observed between the EP values presented in Figure 9 and the BP86-D3/TZ2P interaction energies (Table 10), R = 0.962.

Table 11 presents the energies of the most important orbital–orbital interactions; they correspond to the n(N) → σ_CX_* overlaps (* designates the atibonding orbital here and further in the text). For the A-H∙∙∙B hydrogen bond, the n_B_ → σ_AH_* overlap is the most important orbital–orbital interaction [70,71]. n_B_ designates the lone electron pair of the B proton-accepting centre; σ_AH_* is an antibonding orbital of the A-H bond of the Lewis acid unit. The interaction energy of this overlap is expressed by the following equation (Equation (2)):

ΔE (n_B_→σ_AH_*) = q_i_〈n_B_|F|σ_AH_*〉^2^/(ε (σ_AH_*) − ε (n_B_)) (2)

〈n_B_|F|σ_AH_*〉 is the Fock matrix element, (ε (σ_AH_*) − ε (n_B_)) is the orbital energy difference and q_i_ is the donor orbital occupancy. The n_N_→σ_CH_* overlaps occur for the C-H∙∙∙N intermolecular interactions, i.e., for the hydrogen bonds discussed here in complexes where the hydrogen is the Lewis acid center. For the remaining complexes, the n_N_→σ_CX_* overlaps are observed with X being the halogen center. However, if all centers (X = H, Cl, Br and I) are considered for the n_N_→σ_CX_* overlaps, the following observations can be noted. For the specified substituent, the interaction energy corresponding to the orbital overlap discussed above increases with the increase in the number of CN substituents in the Lewis acid unit. For the specified number of CN substituents for the changing X, there is the following order of the increase in the interaction energy of this overlap Cl < H < Br < I. When the Lewis acid species do not contain CN substituents, the order is slightly different: H < Cl < Br < I.

**Figure 10:**
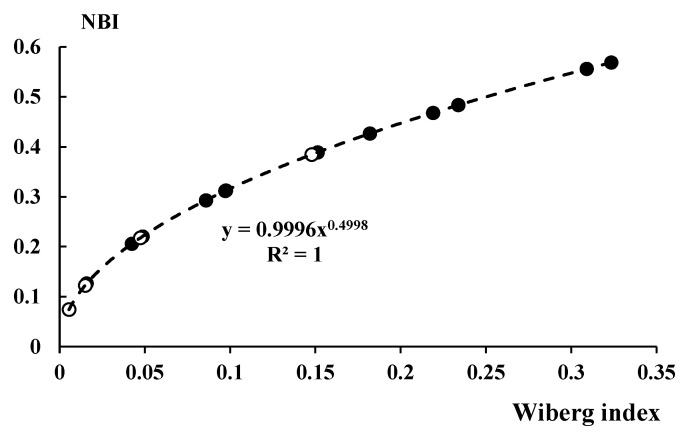
Correlation between NBI and Wiberg indices. Potential regression is applied; all dimers linked by halogen (black circles) and hydrogen (open circles) bonds are taken into account in this correlation.

Table 11 presents also the Wiberg bond indices (WBIs) and the natural binding indices (NBIs) for the X∙∙∙N intermolecular interactions discussed here. The Wiberg index [72,81] corresponds approximately to the bond order, while the NBI is an interaction parameter that can be expressed as the strength (matrix norm) of off-diagonal couplings between the atomic blocks of the natural atom orbital (NAO) density matrix. However, the NBI is also related to the Wiberg index and consequently to the bond order; approximately both indices correspond to the strength of the interatomic link. For all the results presented in Table 11, a linear correlation between the NBI and WBI indices (R^2^ = 0.955) is found. The correlation is even better if the potential function is considered (R^2^ = 1.000), as shown in Figure 10.

**Figure 11:**
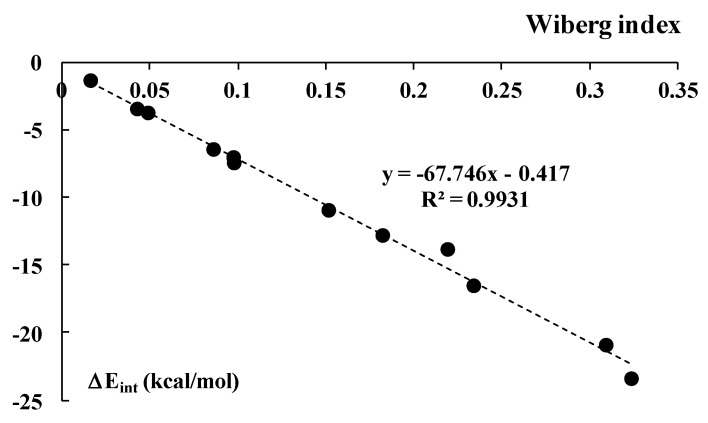
The linear correlation between the Wiberg index and the interaction energy, ΔE_int_ (Equation (1) and Table 10); the hydrogen-bonded systems are excluded from this correlation.

Both WBI and NBI indices correlate with other measures of the interaction strength. In particular, for the halogen-bonded systems, the Wiberg index correlates linearly with ΔE_int_ (Table 10 R^2^ = 0.993, as demonstrated in Figure 11. The systems linked by hydrogen bonds (not included in Figure 11) are excluded from this linear dependence, but show the same tendency, i.e., the increase in WBI index for stronger interactions. A similar linear correlation is observed between NBI and ΔE_int_ (R^2^ = 0.961) when only the halogen-bonded complexes are considered; again, the H-bonded systems do not follow a linear correlation but show the same tendency, i.e., an increase in the NBI index with increasing interaction strength.

## 4. Conclusions

Density functional theory and wave function theory calculations were performed to examine the effects that the electron-withdrawing cyano group CN have on the hydrogen donor ability of methane and the halogen bond donor ability of halomethane. Trimethyl amine was used throughout as the hydrogen or the halogen bond acceptor. Specifically, the equilibrium geometries, vibrational frequencies, and BSSE interaction energies for the complexes CH_3−n_(CN)_n_X∙∙∙NMe_3_ (n = 0–3; X = H, Cl, Br, I) were investigated.

The computational results reveal a substantial and non-additive strengthening of the C-X∙∙∙N interaction with the number of CN groups in the X-bond donor molecule. This interaction strengthening is indicated, for example, by a large increase in the covalent C-X bond, concomitant with a sizeable decrease in the intermolecular X∙∙∙N distance. The increase in the covalent C-X bond brings about a corresponding shift to the red in the corresponding vibrational C-X-stretching frequency. Moreover, direct evidence of the non-additive strengthening of the C-X∙∙∙N bond is provided by changes in the BSSE interaction energies per CN group, relative to the interaction when there are no CN groups (n = 0). Both the strength and the nature of the C-X∙∙∙N bond interactions as a function of the number of CN groups were examined within the framework of the atoms in molecules theory.

Further evidence of the enhancement effects of the CN group on the C-X∙∙∙N bond is given by the results of both the energy decomposition and the NBO analyses. Particularly, the absolute values of all interaction energy terms increase with the increase in the number of CN groups for the specified X-center. The electrostatic interactions accompanied by the electron charge shifts follow the increase in the Pauli repulsion with the decrease in the C-X∙∙∙N bond length, since the sum of attractive interaction energies correlates with the repulsion interaction energy. The electrostatic interaction is the most important attractive term for all complexes, except for the species where methane acts as the Lewis acid unit where the dispersion interaction plays the crucial role. For the majority of complexes investigated here, the next important factor is the orbital–orbital interaction energy; only for the weakest interactions is the dispersion more pronounced than orbital interaction. The NBO approach shows that the n→σ* orbital–orbital overlap is a signature of both hydrogen and halogen bonds. This kind of interaction is strengthened for the specified X-center with the increase in the number of CN groups.

## Figures and Tables

**Table 1:** Optimized geometries for the CH_3−n_(CN)_n_H∙∙∙NMe_3_ complexes; C-H bond lengths, H⋅⋅⋅N distances and C-H⋅⋅⋅N angles are given.

n	C-H (Å)	H∙∙∙N (Å)	C-H∙∙∙N (°)
ωB97X-D/aug-cc-pVTZ			
0	1.089	2.595	151.2
1	1.094	2.326	151.0
2	1.108	2.067	157.2
3	1.151	1.765	180.0
MP2/aug-cc-pVTZ			
0	1.088	2.569	155.8
1	1.092	2.370	145.1
2	1.106	2.066	153.2
3	1.159	1.711	180.0
MP2/aug-cc-pVDZ			
0	1.099	2.522	158.3
1	1.104	2.292	151.8
2	1.118	2.043	154.5
3	1.172	1.693	180.0

**Table 2:** Optimized geometries for the CH_3−n_(CN)_n_Cl∙∙∙NMe_3_ complexes; C-Cl bond lengths, Cl⋅⋅⋅N distances and C-Cl⋅⋅⋅N angles are given.

n	C-Cl (Å)	Cl∙∙∙N (Å)	C-Cl∙∙∙N (°)
ωB97X-D/aug-cc-pVTZ			
0	1.787	3.321	168.6
1	1.789	3.018	175.5
2	1.804	2.810	178.9
3	1.838	2.620	180.0
MP2/aug-cc-pVTZ			
0	1.781	3.115	169.1
1	1.785	2.896	171.1
2	1.804	2.679	177.5
3	1.876	2.385	180.0
MP2/aug-cc-pVDZ			
0	1.798	3.116	169.5
1	1.803	2.903	171.7
2	1.823	2.700	177.8
3	1.888	2.439	180.0

**Table 3:** Optimized geometries for the CH_3−n_(CN)_n_Br∙∙∙NMe_3_ complexes; C-Br bond lengths, Br⋅⋅⋅N distances and C-Br⋅⋅⋅N angles are given.

n	C-Br (Å)	Br∙∙∙N (Å)	C-Br∙∙∙N (°)
ωB97X-D/aug-cc-pVTZ			
0	1.941	3.102	179.9
1	1.953	2.911	179.2
2	1.981	2.725	179.7
3	2.046	2.506	180.0
MP2/aug-cc-pVTZ			
0	1.933	2.896	179.5
1	1.951	2.711	178.7
2	1.997	2.505	179.3
3	2.099	2.302	180.0
MP2/aug-cc-pVDZ			
0	1.951	2.925	180.0
1	1.969	2.747	179.2
2	2.013	2.548	179.5
3	2.106	2.347	180.0

**Table 4:** Optimized geometries for the CH_3−n_(CN)_n_I∙∙∙NMe_3_ complexes; C-I bond lengths, I⋅⋅⋅N distances and C-I⋅⋅⋅N angles are given.

n	C-I (Å)	I∙∙∙N (Å)	C-I∙∙∙N (°)
ωB97X-D/aug-cc-pVTZ			
0	2.145	3.099	179.9
1	2.171	2.890	180.0
2	2.217	2.715	180.0
3	2.300	2.536	180.0
MP2/aug-cc-pVTZ			
0	2.138	2.875	179.9
1	2.170	2.697	179.7
2	2.222	2.543	179.5
3	2.292	2.424	180.0
MP2/aug-cc-pVDZ			
0	2.164	2.889	179.9
1	2.198	2.710	179.8
2	2.248	2.565	179.7
3	2.310	2.457	180.0

**Table 5:** Stretching frequencies, ν_C-X_ (cm^−1^), for CH_3−n_(CN)_n_X∙∙∙NMe_3_ (X = H, Cl, Br, or I; n = 0–3).

n	ν_C-H_	ν_C-Cl_	ν_C-Br_	ν_C-I_
ωB97X-D/aug-cc-pVTZ				
0	3033	748	632	556
1	3024	762	452	418
2	2846	493	411	358
3	2210	465	345	298
MP2/aug-cc-pVDZ				
0	3045	747	624	542
1	3034	747	427	389
2	2854	473	374	340
3	2160	387	308	302

**Table 6:** BSSE-corrected MP2//ωB97X-D/aug-cc-pVTZ interaction energies, ΔE(kcal/mol), for CH_3−n_(CN)_n_X∙∙∙NMe_3_ (X = H, Cl, Br, or I; n = 0–3).

n	H	Cl	Br	I
0	−1.47	−1.73	−3.10	−5.41
1	−4.90	−3.28	−5.50	−8.94
2	−8.02	−5.33	−8.89	−13.89
3	−13.17	−8.50	−15.03	−21.49

**Table 7:** BSSE-corrected CCSD(T)//MP2/aug-cc-pVTZ interaction energies, ΔEs (kcal/mol), for CH_3−n_(CN)_n_X∙∙∙NMe_3_ (X = H, I; n = 0–3).

n	H	I
0	−1.46	−4.30
1	−4.69	−7.53
2	−7.49	−12.19
3	−12.21	−18.93

**Table 8:** MP2//wB97X-D/aug-cc-pVTZ topological parameters (a.u.) for CH_3−n_(CN)_n_X∙∙∙NMe_3_ (X = H, Cl, Br, I).

n	ρ_c_	G_c_	V_c_	H_c_	|G_c_/V_c_|
CH_3−n_(CN)_n_H∙∙∙NMe_3_					
0	0.0089	0.0058	−0.0048	0.0010	1.21
1	0.0162	0.0106	−0.0095	0.0011	1.12
2	0.0280	0.0183	−0.0198	−0.0015	0.92
3	0.0550	0.0322	−0.0485	−0.0163	0.66
CH_3−n_(CN)_n_Cl∙∙∙NMe_3_					
0	0.0071	0.0049	−0.0037	0.0012	1.32
1	0.0134	0.0097	−0.0081	0.0016	1.20
2	0.0208	0.0153	−0.0141	0.0012	1.09
3	0.0320	0.0230	−0.0240	−0.0010	0.96
CH_3−n_(CN)_n_Br∙∙∙NMe_3_					
0	0.0137	0.0093	−0.0081	0.0012	1.15
1	0.0200	0.0138	−0.0130	0.0008	1.06
2	0.0296	0.0200	−0.0209	−0.0009	0.96
3	0.0473	0.0302	−0.0372	−0.0070	0.81
CH_3−n_(CN)_n_I∙∙∙NMe_3_					
0	0.0176	0.0110	−0.0104	0.0006	1.06
1	0.0263	0.0166	−0.0175	−0.0009	0.95
2	0.0372	0.0231	−0.0275	−0.0044	0.84
3	0.0900	0.0407	−0.0786	−0.0379	0.52

**Table 9:** Changes in electron density at C-X…N bond critical points (a.u.) per number of CN groups, Δρ_c_/n, present in CH_3−n_(CN)_n_X∙∙∙NMe_3_.

CN Groups	H	Cl	Br	I
1	0.0073	0.0063	0.0063	0.0087
2	0.0096	0.0069	0.0080	0.0098
3	0.0154	0.0083	0.0112	0.0241

**Table 10:** The interaction energy terms (in kcal/mol) and the ΔE_orb_/ΔE_elstat_ ratio for the CH_3−n_(CN)_n_X∙∙∙N(CH_3_)_3_ complexes (X = H, Cl, Br, I).

Lewis Acid Unit	ΔE_int_	ΔE_Pauli_	ΔE_elstat_	ΔE_orb_	ΔE_disp_	ΔE_elstat_/ΔE_orb_
CH_3_H	−1.76	3.56	−1.91	−1.12	−2.29	1.7
CH_2_CNH	−5.60	7.87	−6.56	−3.05	−3.85	2.2
CH(CN)_2_H	−9.52	14.13	−11.47	−7.22	−4.96	1.6
C(CN)_3_H	−17.09	33.66	−23.78	−21.54	−5.44	1.1
CH_3_Cl	−1.31	5.41	−2.65	−1.81	−2.26	1.5
CH_2_CNCl	−3.41	9.38	−6.21	−4.12	−2.47	1.5
CH(CN)_2_Cl	−6.99	17.86	−12.42	−9.75	−2.68	1.3
C(CN)_3_Cl	−13.79	45.54	−28.65	−27.26	−3.41	1.1
CH_3_Br	−3.70	12.95	−8.76	−5.00	−2.89	1.8
CH_2_CNBr	−7.40	22.39	−15.97	−10.41	−3.42	1.5
CH(CN)_2_Br	−12.76	41.85	−28.96	−21.75	−3.91	1.3
C(CN)_3_Br	−20.87	79.17	−51.76	−44.13	−4.14	1.2
CH_3_I	−6.40	22.76	−16.37	−8.74	−4.05	1.9
CH_2_CNHI	−10.90	37.87	−27.52	−16.75	−4.49	1.6
CH(CN)_2_I	−16.48	59.10	−42.28	−28.61	−4.69	1.5
C(CN)_3_I	−23.35	83.70	−58.90	−43.38	−4.77	1.4

**Table 11:** CH_3−n_(CN)_n_X∙∙∙N(CH_3_)_3_ complexes (X = H, Cl, Br, I); the properties of X∙∙∙N contact are given. Q(X)—NBO charge of X; Q(N)—NBO charge of N; WBI—Wiberg bond index; NBI—natural binding index; n→σ* is n(N)→σ_CX_* orbital–orbital interaction energy (in kcal/mol).

Lewis Acid Unit	Q(X)	Q(N)	WBI	NBI	n→σ*
CH_3_H	0.230	−0.348	0.0055	0.0743	0.69
CH_2_CNH	0.283	−0.363	0.0151	0.1227	2.49
CH(CN)_2_H	0.320	−0.367	0.0474	0.2177	8.07
C(CN)_3_H	0.344	−0.344	0.1479	0.3845	29.15
CH_3_Cl	−0.082	−0.339	0.0159	0.1263	0.89
CH_2_CNCl	−0.026	−0.336	0.0424	0.2059	2.22
CH(CN)_2_Cl	0.004	−0.315	0.0972	0.3118	5.02
C(CN)_3_Cl	−0.012	−0.260	0.2190	0.4680	13.65
CH_3_Br	−0.039	−0.335	0.0486	0.2204	3.78
CH_2_CNBr	0.013	−0.325	0.0975	0.3123	6.99
CH(CN)_2_Br	0.040	−0.296	0.1820	0.4267	13.55
C(CN)_3_Br	0.059	−0.250	0.3091	0.5560	27.98
CH_3_I	0.039	−0.337	0.0858	0.2929	6.51
CH_2_CNHI	0.097	−0.326	0.1513	0.3890	11.84
CH(CN)_2_I	0.143	−0.308	0.2338	0.4835	19.74
C(CN)_3_I	0.187	−0.290	0.3235	0.5688	30.21

## Data Availability

Not applicable.
